# The Opportunities and Outlook

**Published:** 1920-09-04

**Authors:** 


### THE PUBLIC HEALTH SERVICE.

### The Opportunities and Outlook.

There is a general movement on the part of medical as well as public opinion to give preventive medicine its proper place, not only in the medical curriculum, but also in the medical services. This is all to the good, and as a branch of preventive medicine the Public Health Service is naturally sharing in this appreciation. But although medicine in the past was almost solely concerned with the curing, as distinct from the prevention of disease, and while we welcome the swing of the pendulum from the former to the latter, yet we must guard against the swing going too far and resulting in a final retardation of prevention. The new phase will after all take no sound hold until the public has fully grasped its significance, and this position will not be reached quite as rapidly as some too enthusiastic reformers imagine

### The Need for Caution.

In the next place, we must beware of too much specialism in public health as in other medical services. We must, of course, admit that the perfection to which some branches of medicine and surgery have been brought is largely through specialism, but that does not do away with the fact that specialists tend to become narrow and one-sided, and the Public Health Service is just as likely to suffer in this respect as any other. The question is how to prevent this, and a solution, we think, will be found in the method of education of the specialist. First, it should be taken as an axiom that in studying medicine a man must study both health and disease, and the further question then arises as to how far the present medical curriculum gives opportunity of a thorough acquaintance with both. If you desire an answer you will get a different one according to whether you ask a public health officer or a general practitioner. The former will say without hesitation that the student is taught much too little about health and the latter that he learns far too little about disease. The man who has had experience of public health work *and* general practice will say that both are correct. With this last view we must perforce agree, for all that the medical curriculum can be expected to do is to lay a solid scientific foundation for whichever branch of medicine a man intends to make his life-work.

### After Qualification.

After qualification, the next thing is to take up some hospital appointments in different branches of medicine, and during this period, if the student has any administrative bent or for any reason thinks that he would like the Public Health Service, he may study for and take his D.P.H. If after taking this qualification he still thinks his inclinations are towards this branch of preventive medicine, he would be wise to gain some experience in general practice before specialising in public health, but as to the wisdom of this we are aware that there is sharp difference of opinion among medical officers of health. In our opinion, if narrowness and overspecialisation are to be avoided in the Public Health Services, every health officer should know something of private practice and private practitioners. This experience can be gained only by a year or two's work as an assistant, or in practice for oneself.

### The Clinical and Administrative Sides.

After this period the next point to be decided is whether the candidate desires to take up the administrative or the clinical and scientific side of public health, and one must admit that the best-paid posts are administrative, but these must be obtained through a subordinate post of assistant M.O.H. The days are passed when the higher posts could be obtained without actual previous experience in administrative work, and this is as it should be. There is now a tendency to give the whole-time M.O.H. posts to those who have been assistant M.O.H.s in the administrative work. These posts, in our opinion, should be open to school, tuberculosis, and other subordinate public health officers, provided they are whole-time assistants in a Public Health Department, otherwise such posts as school medical officers and tuberculosis officers and their assistants will remain, as many of them are now, "blind alley" occupations, to the detriment of these particular services and the Public Health Services generally. Even now these two particular services have difficulty in getting good men, for a newcomer either looks for an administrative post or else avoids the Public Health Services altogether.

There are indications, however, that authorities are coming to realise that all these subordinate Public Health Services must be manned by officers having the status of assistant M.O.H.s, and that in the not distant future these services will cease to be Cinderellas and blind-alleys to budding M.O.H.s, but will act as portals to the best posts of administrative work. It is not, therefore, wise, should there be any dearth of assistantships in administrative posts for a holder of the D.P.H. to refuse an appointment as tuberculosis or school medical officer, provided that they form an actual branch of the Public Health Department of a County, County Borough, or other large Sanitary Authority.

To sum up, therefore, we advise the newly qualified man seeking to enter the public health service to (1) Have a good general knowledge of medicine in *all its branches*; (2) Take a post of an assistant M.O.H., or failing this, tuberculosis or other subordinate officer, provided they are part of the Public Health Department, and the officer is given the status of an assistant M.O.H.; (3) Make himself familiar with the general public health work of the district in which he is situated; and (4) If he prove a success he need not fear but that his turn for one of the higher posts will come in due time.

